# Structural Evolution of Chemically-Driven RuO_2_ Nanowires and 3-Dimensional Design for Photo-Catalytic Applications

**DOI:** 10.1038/srep11933

**Published:** 2015-07-07

**Authors:** Joonmo Park, Jae Won Lee, Byeong Uk Ye, Sung He Chun, Sang Hoon Joo, Hyunwoong Park, Heon Lee, Hu Young Jeong, Myung Hwa Kim, Jeong Min Baik

**Affiliations:** 1 School of Materials Science and Engineering, KIST-UNIST-Ulsan Center for Convergent Materials, Ulsan National Institute of Science and Technology (UNIST), Ulsan 689-798, Republic of Korea; 2 Department of Chemistry & Nano Science, Global Top 5 Program, Ewha Womans University, Seoul 120-745, Republic of Korea; 3 School of Energy & Chemical Engineering Department of Chemistry Ulsan National Institute of Science & Technology (UNIST), Ulsan 689-798, Republic of Korea; 4 School of Energy Engineering, Kyungpook National University, Daegu 702–701, Republic of Korea; 5 Department of Materials Science and Engineering, Korea University, Anam-dong 5-ga, Seongbuk-gu, Seoul 136-713, Republic of Korea; 6 UNIST Central Research Facilities (UCRF), Ulsan National Institute of Science and Technology (UNIST), Ulsan 689-798, Republic of Korea

## Abstract

Growth mechanism of chemically-driven RuO_2_ nanowires is explored and used to fabricate three-dimensional RuO_2_ branched Au-TiO_2_ nanowire electrodes for the photostable solar water oxidation. For the real time structural evolution during the nanowire growth, the amorphous RuO_2_ precursors (Ru(OH)_3_·H_2_O) are heated at 180 ^°^C, producing the RuO_2_ nanoparticles with the tetragonal crystallographic structure and Ru enriched amorphous phases, observed through the in-situ synchrotron x-ray diffraction and the high-resolution transmission electron microscope images. Growth then proceeds by Ru diffusion to the nanoparticles, followed by the diffusion to the growing surface of the nanowire in oxygen ambient, supported by the nucleation theory. The RuO_2_ branched Au-TiO_2_ nanowire arrays shows a remarkable enhancement in the photocurrent density by approximately 60% and 200%, in the UV-visible and Visible region, respectively, compared with pristine TiO_2_ nanowires. Furthermore, there is no significant decrease in the device’s photoconductance with UV-visible illumination during 1 day, making it possible to produce oxygen gas without the loss of the photoactvity.

Three-dimensional (3D) branched nanowire arrays have received much attention owing to their peculiar and interesting optical, electronic, and catalytic properties^1^,^2^,^3^. Because of their very high surface-to-volume ratios, the direct conduction pathway for charge transport, and the low light reflectance, the 3D nanostructures can in principle are used to create very practical nanophotonic devices such as photochemical cells, sensors, light-emitting diodes, and solar cells^3^,^4^,^5^.

Ruthenium dioxide (RuO_2_) are of great interest as a promising candidate as electrodes in electrochemical devices and active catalysts due to extremely low resistivity, excellent chemical and thermal stability, catalytic property^6^,^7^. In particular, it is well-known to be among the most efficient electrocatalysts for the oxygen evolution reaction (OER) in alkaline and acid electrolytes, promoting the photoelectrochemical (PEC) water oxidation in photochemical cells. The sluggish anodic OER as well as the predominant absorption in the UV region may be a main factor in determining the capability of the titanium dioxide (TiO_2_)-based photoelectrodes^8^. Recently, the IrO_2_/hemin-coated TiO_2_ nanowires showed much enhanced photocurrent with ~100% increase compared to the pristine TiO_2_ nanowires^9^. The photocatatytic activities of TiSi_2_/graphene nanoparticles and Nb/N-doped anatase TiO_2_ nanoparticles were reported to be improved by the loading of RuO_2_ nanoparticles^10^,^11^. Also, the RuO_2_-Au composites enhanced the catalytic property with ~30% than pure Au^12^. Thus, most of the catalysts are formed as a form of fine nanoparticles. The photocatalytic activity may be enhanced by the advantages imparted by the 3D branched nanostructure, as mentioned above. Furthermore, since 2012, it is also interesting that the price of Ru has decreased by approximately 40% on sales in Asia and U.S.^13^.

In spite of its great potential toward future real applications, on the other hand, the growth of highly single crystalline RuO_2_ nanowires has been recently reported by thermal oxidation or chemical vapor deposition (CVD) of appropriate Ru-based precursors and reactive sputtering using pure Ru metal targets^7^,^14^,^15^. However, it is still challenging to make RuO_2_ nanowires with well-defined crystal structures with desirable density in real applications. For the RuO_2_ nanowires grown by a CVD, a process referred to as vapor-solid (VS) growth model was suggested via the formation of gaseous RuO_4_ species which is a highly volatile and very low melting point intermediate^14^. A thermal conversion from amorphous oxides nanoparticle precursor was also suggested to synthesize single crystalline nanowires of oxide materials^15^. However, it is necessary to explore this mechanism in greater detail in order to fully demonstrate the preferential and unidirectional crystal growth of RuO_2_.

Here, we focus on the real-time structural evolution of chemically-driven RuO_2_ nanowires for the growth mechanism and report a facile method to fabricate RuO_2_ branched Au-TiO_2_ nanowire arrays for the photostable solar water oxidation. The in-situ synchrotron x-ray diffraction (in-situ SXRD) shows the direct transition of the amorphous RuO_2_ precursors (Ru(OH)_3_·H_2_O) to crystalline RuO_2_ nanoparticles with the tetragonal crystallographic structure at 180 ^°^C, without the intermediate change formation. In the high-resolution transmission electron microscope (HRTEM), we find that most of the crystallized nanoparticles are in the vicinity of the nanowires and the amorphous region is Ru enriched by about 10% in comparison to the RuO_2_ nanowire. This means that the growth proceeds by Ru diffusion to the nanoparticles, increasing the size of the nanoparticles, eventually, forming the nanowires. At low temperature less than 250 ^°^C, the diffusion is so slow, thereby, the nanowires are very short (less than 1 μm at the growth time of several hours). We believe that this process is supported by the nucleation theory. We also carefully explore the photocatalytic performance of the water splitting of the branched nanowires and the performance is compared with pristine TiO_2_ nanowires. We found that the photoactivity was effectively enhanced in the entire UV-visible region, especially more effective in visible light, due to the high catalytic properties of the RuO_2_ for the water oxidation and the efficient plasmonic absorption with efficient charge separation to TiO_2_ and Au interface, catalyzing the oxygen evolution of the 3D RuO_2_ branched structure on the Au surface. The electrodes were quite stable during long-term use (we believe that it may ascribe to the high-crystalline properties of the RuO_2_ nanowires).

## Results and Discussion

**Figure 1:**
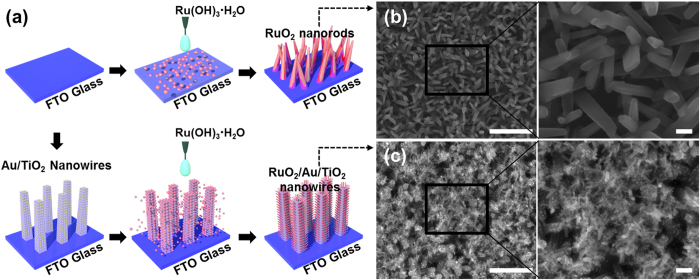
(**a**) Schematic diagram for the fabrication of RuO_2_ nanowires and 3D RuO_2_ branched Au-TiO_2_ nanostructure, (**b**,**c**) top-view SEM images of RuO_2_ nanowires and 3D branched nanostructures (scale bars in the left and right images, 1 μm and 100 nm, respectively).

The schematic diagrams outlining the fabrication process of 1D RuO_2_ nanowires and 3D RuO_2_ branched Au-TiO_2_ nanowires are shown in Fig. 1a and detailed information described in Experimental section. Scanning electron microscopy (SEM) images in Fig. 1b shows the product (of a reaction carried out at 250 ^°^C) to consist of long, randomly oriented RuO_2_ nanowires without any catalyst particles at the end of their tips. The nanowires (0.5 ~ 1 μm long and 130 ~ 170 nm in diameter) grow out of the plane of the substrate in the rectangular shape. Figure 1c shows RuO_2_ nanowires directly grown on hydrothermally-grown TiO_2_ nanowires with Au nanoparticles (AuNPs), producing 3D branched nanowires. The lengths of most of the nanowires fall in the range 60 ~ 80 nm. The mean nanowire width was determined to be 25 nm. The smaller RuO_2_ nanowires in the 3D branched structures ascribes to the decrease in the amount of the precursor, which is an important factor in determining the length and width of the nanowires.

**Figure 2:**
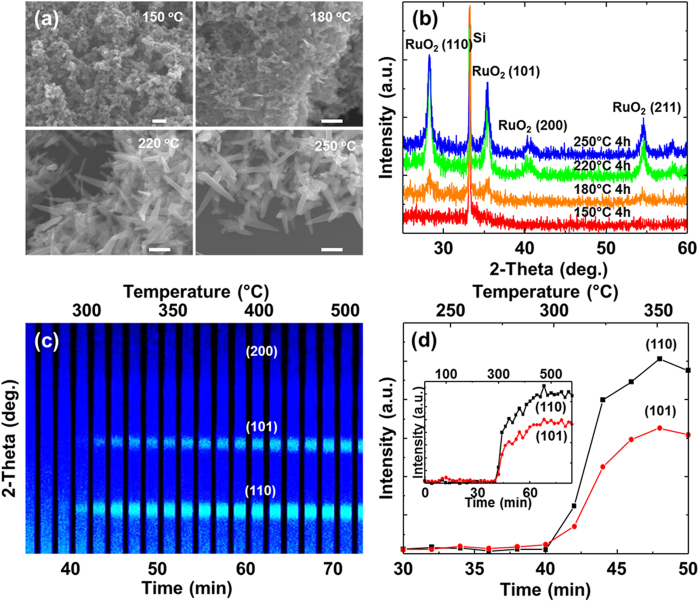
(**a**) SEM images and (**b**) the corresponding XRD patterns (● : (110), ○ : (101), ■ : (200), and □ : (211)) of RuO_2_ nanowires as a function of temperature (scale bar, 100 nm). The peak at 33.2 ^°^ is due to the substrate. (**c**) The in-situ synchrotron radiation diffraction from the growth of RuO_2_ nanowires as the temperature increases to 150 ~ 600 ^°^C. Each vertical line is one XRD scan, with the intensity represented by the color. The first scan is at the left, and time progresses right. (**d**) The change in the intensity of (101) and (110) plane with time and inset image is full time.

Figure 2a shows SEM images of the as-grown RuO_2_ nanowires on a Si substrate synthesized by heating the RuO_2_ precursors in air atmosphere in the range of 150 ~ 250 ^°^C during 4 hrs. At low temperature less than 150 ^°^C, only nanoparticles were observed and there were no nanowires on the substrates. In ex-situ x-ray diffraction (XRD), there are no peaks in the spectra, meaning that the nanoparticles are still amorphous. As the temperature increases to 180 ^°^C, three peaks corresponding to the (110), (101), and (211) planes were clearly observed, confirming that the amorphous nanoparticles start to be crystallized at the temperature. In the SEM image, many straight nanowires with apparent tapering at the end and without any cluster at the tip were also seen. Further increase in the temperature enhances the growth of the nanowires, increasing the intensity of the three peaks, and produces additional peak corresponding to the (200) plane. At 250 ^°^C, the length and diameter of the nanowires are in the range of 150 nm and 18 nm, respectively. The length and diameter of the nanowires increase as the temperature increases, while the aspect ratio decreases with the temperature (Supplementary Fig. S1).

In order to further investigate the synthetic process, such as intermediate phase formation over annealing time and temperature, in-situ SXRD experiments were carefully performed in the course of the growth process. In-situ SXRD data provides direct identification of the solids and a sequence of events in the formation of different objects and their growth. The ruthenium hydroxide precursors are put on the stages and then heated by external power sources up to 600 ^°^C with the ramping speed of 7 ^°^C/min. 42 data points for 2 minutes at each temperature interval were obtained. However, experience tells that the measured temperature is not equal to that of the nanoparticles, which will be lower. In the initial stage of the heating, there is no peak. As the temperature increases to about 300 ^°^C, two peaks corresponding to the (110) and (101) planes are observed. However, we could observe any other peaks, indicating the spontaneous growth of the RuO_2_ phases from the amorphous phases without any intermediate phase formed.

The above spontaneous growth of the nanowires during the oxidation of precursors without the use of conventional templates or catalysts has received significant attention in the nanotechnology community. The VS model was usually proposed to understand the growth of ZnO and SnO_2_ nanowires based on the evaporation of the precursors^16^,^17^. However, the RuO_2_ synthesis is carried out at low temperature of ~180 °C, at which the equilibrium vapor pressures of the pure elements are negligibly small, therefore cannot be ascribed to the model. The growth of the nanowires may ascribe to the diffusion of the cations during the oxidation, forming the tip-like geometry of a single nanowire, in which the driving force for the diffusion is related to the internal stress associated with the phase formation at the CuO/Cu_2_O interface. Also, metal-oxides nanowires such as VO_2_, V_2_O_5_, MoO_2_, MoO_3_, and Fe_3_O_4_, can be grown via the diffusion and solidification of nanodroplets near the bulk melting point^18^,^19^,^20^,^21^,^22^,^23^.

**Figure 3:**
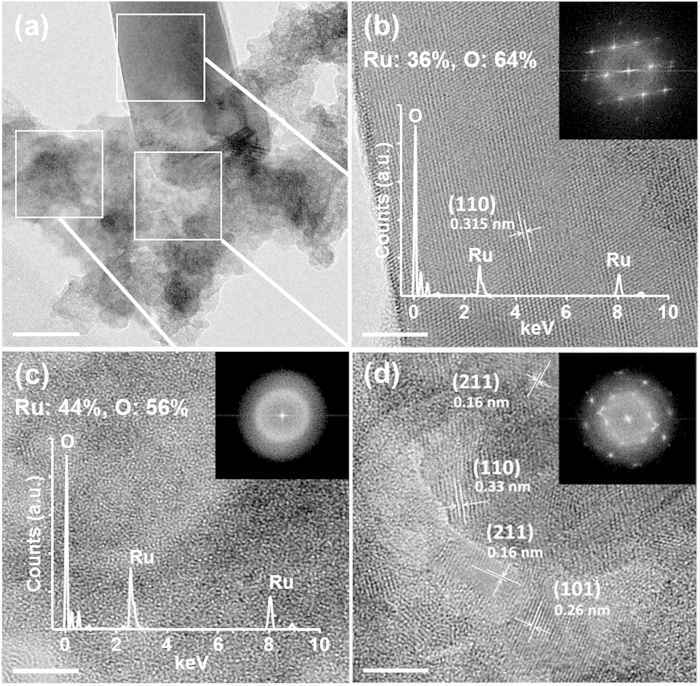
(**a**) BF- TEM image near the bottom of the RuO_2_ nanowire (scale bar 20 nm). (**b**) HRTEM image of RuO_2_ nanowire obtained after annealing at 190 ^°^C for 1 hr. (**c**) Magnified HRTEM image of amorphous RuO_2_ region and (**d)** crystalline RuO_2_ nanoparticles at the interface between the region and nanowire (Fig. b, c and d scale bar 5 nm). Inset in (**b**,**c**) show the EDS spectra.

The detailed growth mechanism was characterized by bright-field TEM (BFTEM) and HRTEM images, as shown in Fig. 3. Figure 3a is a BFTEM image of a single nanowire grown at 190 °C with a diameter of approximately 30 nm. The HRTEM image and the corresponding fast-Fourier transformed (FFT) of the nanowire reveal highly ordered lattice fringes, demonstrating that the nanowire is a defect-free single crystal (Fig. 3b). The RuO_2_ nanowire has identified as a tetragonal crystalline (110) phase. In the vicinity of the region where the nanowire starts to be grown, there are some crystallized RuO_2_ nanoparticles that are ~4 nm in size are clearly seen in the vicinity of the nanowires, while most of the nanoparticles exist as a form of amorphous elsewhere (Fig. 3c,d). Any additional Ru-based crystalline phases except the RuO_2_ phase, tetragonal crystallographic structure, are not observed. This implies that the nanowires are grown from the amorphous nanoparticles by the direct recrystallization process.

Previously, it was reported that the amorphous RuO_2_ precursors started to lose the water of the hydration to create the anhydrous RuO_2_ nanoparticles and Ru metals at 90 °C, as follows^24^;


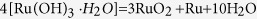


The rate of Ru/RuO_2_ content was approximately 0.3 in N_2_ ambient, meaning that the nanoparticles are amorphous, and increased with the annealing temperature. Actually, it was observed that the amorphous region in Fig. 3c, about 20 nm away from the nanowire, was Ru enriched by about 10% in comparison to the RuO_2_ nanowire in Fig. 3b, determined by energy dispersive spectroscopy (EDS). However, most of nanoparticles are seen to be crystalline in the vicinity of the nanowire. The size of the nanoparticles is in the range of 2 ~ 4 nm. According to the nucleation theory, the critical radius (r*) of the nucleation critically depends on the surface energy (*γ*), the latent heat of fusion (*ΔH*_*f*_), the melting temperature (*T*_*m*_), and the growth temperature (*T*_*g*_), which is given by,


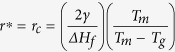


Here, the melting temperature and latent heat of fusion are 1200 ^°^C and 1.66 × 107 J/m^3^, respectively. The surface free energy of (110) and (001) planes are 1.136 J/m^2^ and 1.398 J/m^2^, respectively^24^,^25^. In this case, the critical thickness of RuO_2_ is calculated to be about 2 nm at 190 ^°^C, similar with those observed in the TEM images (Fig. 3d). The observation of the RuO_2_ crystalline nanoparticles between the amorphous phase and the nanowire may suggests that the amorphous phase is crystallized before the growth of the nanowires.

The growth rate of the nanowires is mainly limited by the rate of transport of mass, the diffusion rate of Ru and RuO_2_ materials produced by the heating process of RuO_2_ precursor. The activation energy (ΔE) for the migration of Ru and RuO_2_ of ΔE is supposed to be 384 kJ/mol and 3140 kJ/mol, respectively through latent heat of fusion (Ru: 24 kJ/mol, RuO_2_: 314 kJ/mol)^26^. This indicates that ΔE of RuO_2_ is about 8 times of magnitude of larger than that of Ru, meaning that the diffusion of Ru metals is the dominating process. The concentration gradient of Ru from the amorphous phase to the nanowires promotes the diffusion, enhancing the growth of the nanowires. We also believe that the Ru metals migrate from the bottom to top region of the nanowires through the surface because the bulk diffusion becomes significant at only high temperature. In O_2_ ambient, the Ru reacts with oxygen, producing the RuO_2_ on the surface^27^. Actually, at free oxygenless atmosphere such as ambient gases such as nitrogen or under vacuum conditions, there are no nanowires on the substrates.

**Figure 4:**
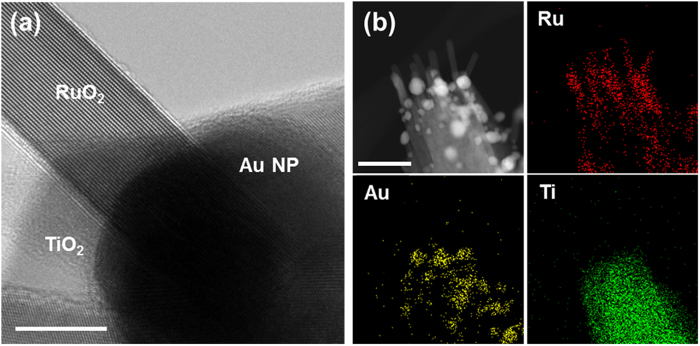
(**a**) TEM image of RuO_2_ nanowire on Au nanoparticle-decorated TiO_2_ nanowire. It is clearly seen that the nanowire is grown on the Au nanoparticle (scale bar 20 nm). (**b**) High-angle annular dark field (HAADF) scanning TEM image of 3D branched nanowires. EDS elemental distribution map of Ru, Au, and Ti (scale bar 100 nm).

The RuO_2_ nanowires were successfully grown on the Au-decorated TiO_2_ nanowires, by precisely controlling the concentration of precursors and the growth temperature. HRTEM image in Fig. 4a shows that single-crystalline RuO_2_ nanowire is directly grown on the Au nanoparticle. EDS elemental mapping of Fig. 4b clearly also confirms each elemental distribution.

**Figure 5:**
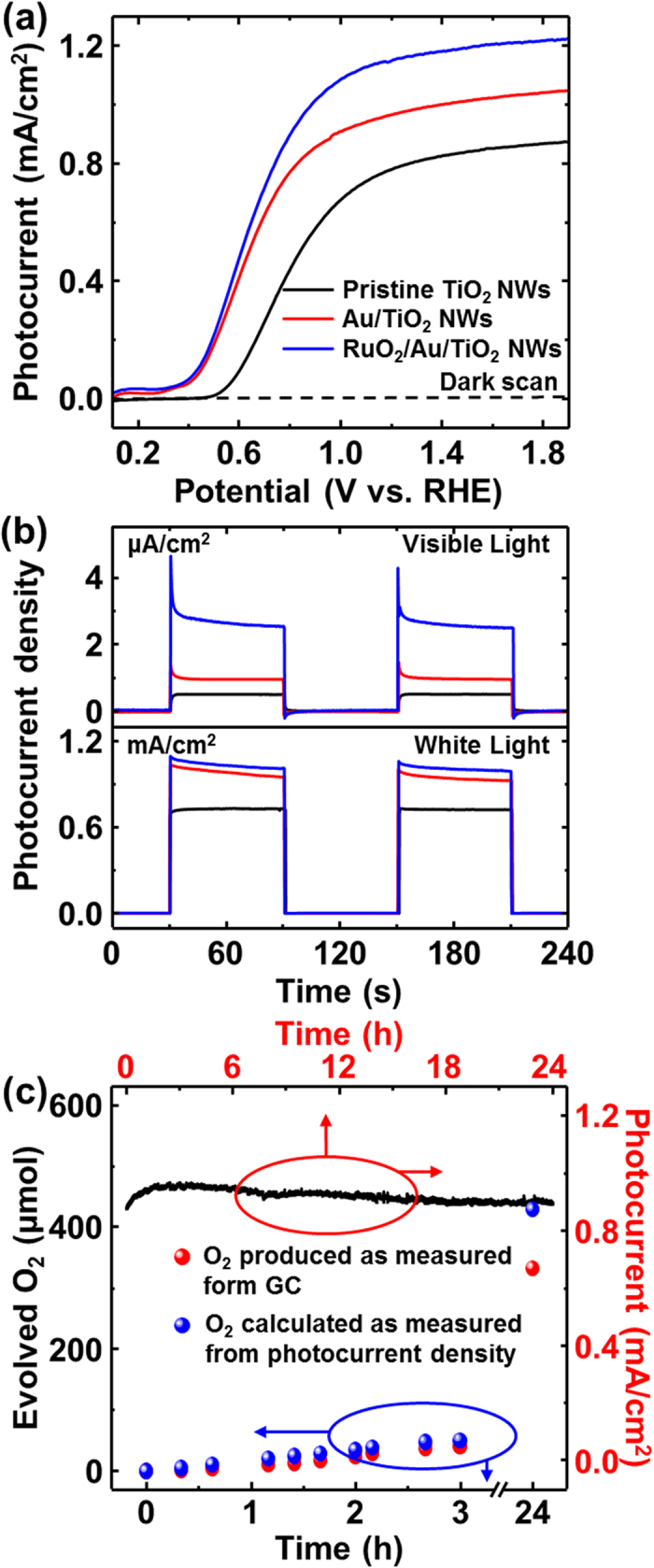
(**a**) Photocurrent versus potential characteristics of 0.5 M Na_2_SO_4_ (pH 7.2) electrolyte under AM 1.5 light illumination measured against the Ag/AgCl electrode with 30 mV/s scan rate. (**b**) Photocurrent density for chopped full spectrum (AM 1.5G) and visible illumination (λ > 420 nm long pass filter with AM 1.5G) at 1.62 V vs. RHE. (**c**) The quantity of evolved oxygen (red dots) measured (gas-chromatographically) as a function of time. The photocurrent simultaneously recorded with light illumination of white light (AM 1.5G) at 1.23 V vs. RHE.

The PEC performance of the TiO_2_-based photoelectrodes was examined in a three-electrode configuration with Ag/AgCl as the reference electrode and a Pt wire as the counter electrode. Figure 5a shows the photocurrent-potential characteristics of the TiO_2_ nanowires, AuNPs decorated TiO_2_ nanowires, and RuO_2_ branched AuNPs-TiO_2_ nanowires recorded in 0.5 M Na_2_SO_4_ electrolyte (pH = 7.2) under AM 1.5G illumination of 100 mW/cm^2^. The dark scans revealed a small background current of ~2 × 10^−3^ mA/cm^2^, negligible compared to the photocurrent densities of all photoelectrodes. Upon illumination with white light, the pristine TiO_2_ nanowires electrodes show a photocurrent density of 0.673 mA/cm^2^ at 1.23 V vs. RHE (0.61 V vs. Ag/AgCl). As AuNPs are decorated onto the nanowires, the photocurrent density increases to 0.909 mA/cm^2^ at the same potential, associated with the surface plasmonic resonance effect of the AuNPs^28^. It was also observed that under visible-light illumination, the photocurrent density of AuNPs-TiO_2_ nanowires was increased by 1.93 times, in comparison with pristine TiO_2_ nanowires, obtained by adding a 420 nm long-pass filter to the white source, as shown in Fig. 5b. The formation of RuO_2_ nanowires on AuNPs-TiO_2_ nanowires increases the photocurrent density to 1.052 mA/cm^2^, around 56% enhancement, compared with pristine TiO_2_ nanowires. It is believed that the RuO_2_ promotes the electron transfer to the AuNPs due to the high catalytic properties for the water oxidation and the efficient hole injection to RuO_2_ to AuNPs, catalyzing the oxygen evolution (Supplementary Fig. S2). This process is prominent seen under visible light. We also observed that the photocurrent of the branched nanowires significantly increased over approximately 0.7 V vs. RHE, compared to that of the AuNPs-TiO_2_ nanowires, with a slight shift in the onset voltage. This may be related to the flat band voltage as 0.41 eV of the work function difference between Au and RuO_2_. The slight shift in the onset voltage may also be ascribed to the low overpotential on the Au-TiO_2_ nanowires associated with the oxygen reduction^29^,^30^.

For the long photostability of the electrodes, the photocurrent was measured under illumination conditions of illuminated with white light at 1.23 V vs. RHE as a function of time up to 24 hrs. Figure 5c shows that the photocurrent decreases slowly up to 24 hrs after a little increase in photocurrent within 3 hrs, which may ascribe to the build-up of hydrogen concentration on Pt electrodes. The rapid decrease in the photocurrent of the efficient photoelectrodes as major limitation to commercialization was not observed here^31^, which may ascribe to the highly crystalline properties of the RuO_2_ nanowires and the good chemical stability of the TiO_2_. Gas chromatographic measurements under the illumination conditions described above also shows that there is no significant difference between the calculated oxygen amount from the photocurrent density and the oxygen evolution amount. This may imply that the RuO_2_ branched Au-TiO_2_ electrodes has stoichiometric amount reaction for H_2_ and O_2_ evolution and avoid the back reaction, showing quite stable performance during the gas evolution.

## Conclusion

We report the chemically-driven RuO_2_ nanowires and a facile method to fabricate three-dimensional RuO_2_ branched Au-TiO_2_ nanowire arrays for the photostable electrodes in PEC water oxidation. The amorphous RuO_2_ precursors (Ru(OH)_3_·H_2_O) are heated at 180 ^°^C, producing the RuO_2_ nanoparticles with the tetragonal crystallographic structure and Ru enriched amorphous phases, observed by the real time structural evolution during the growth process through the in-situ SXRD and the HRTEM images. Growth proceeds by Ru diffusion to the nanoparticles, followed by diffusion to the growing surface of the nanowire in oxygen ambient, supported by the nucleation theory. The RuO_2_ branched Au-TiO_2_ nanowire arrays shows a remarkable enhancement in the photocurrent density by approximately 60% and 200%, in the UV-visible and Visible region, respectively, compared with pristine TiO_2_ nanowires at the same potential. Furthermore, there is no significant change in the device’s photoconductance with UV-visible illumination during 1 day, making it possible to produce oxygen gas without the loss of the photoactivity. This may ascribe to the highly crystalline properties of the RuO_2_ nanowires and means the good photostability of the designed electrode.

### Methods

### Synthesis of RuO_2_ nanowires and Growth Mechanism

Highly-crystalline RuO_2_ nanowires were grown by a simple annealing process of the RuO_2_ precursor via a simple reaction between RuCl_3_·xH_2_O and NaOH by carefully controlling pH (~9.00) of the aqueous solution at room temperature, as described previously^14^. This was found to be so simple and facile method for the growth of the nanowires. During this process, the amorphous RuO_2_ precursor (Ru(OH)_3_·H_2_O) were washed several times with deionized (DI) water to remove the remaining chlorides and sodium hydroxide, followed by the redispersion in DI water. For the detailed growth mechanism, it was spread on a Si (001) wafer and after drying at room temperature for 2 hrs, the sample are heated at the temperature ranging from 150 to 250 ^°^C for 4 hrs.

### Growth of 3D Branch-Shaped Nanostructure with Au nanoparticles

For the efficient oxygen production, TiO_2_ nanowire arrays were first grown on fluorine-doped tin oxide (FTO) substrate by the hydrothermal method, reported elsewhere^32^. After washing with DI water, the sample was annealed in air at 450 ^°^C for 4 hrs to improve the crystallinity of the nanowires. The AuNPs are formed by metal aggregation. The ~1 nm thick (mass thickness) Au film was then deposited on the TiO_2_ nanowires using e-beam evaporation at a very slow deposition rate of less 0.1 Å/s and annealed at the temperature 250 ^°^C for 1 min in nitrogen gas. The base pressure was maintained at 2.0 × 10^−6^ Torr. It produced well-separated AuNPs covering the nanowire uniformly. Finally, the RuO_2_ precursor mixed in DI water (RuO_2_ precursor are well-controlled from 3 × 10^−6^ to 6 × 10^−4^ wt%) are dropped into the sample and dried at 2 hrs, followed by the annealing process in atmosphere.

### Microstructural analysis

The real time structural evolution during the growth process was done by the in-situ synchrotron radiation diffraction as the temperature increases to 150 ~ 600 ^°^C, which was carried out at the 3D beamline at Pohang Accelerator Laboratory (PAL). For high-resolution XRD measurements, the wavelength of the incident X-ray was set at 1.488 Å by a double-bounce Si (111) monochromator. The SEM was done using a PHILIPS XL30S with an accelerating voltage of 5 kV. The HRTEM images were collected using a Cs-corrected JEM-2100 operated at 200 kV.

### Photoelectrochemical Characterization

The PEC properties of the electrodes were studied in a three electrode cell with Ag/AgCl as the reference electrode and a Pt wire as the counter electrode. The working electrode area is in the range of ~0.2 cm^2^. 0.5 M Na_2_SO_4_ aqueous solution (pH = 7.2) was used as an electrolyte for PEC measurements. Linear sweeps and time-profiled current generation were obtained by an IviumStat electrochemical analyzer, with Ag/AgCl as reference and Pt wire as counter electrode under simulated sunlight with a 150 W xenon lamp coupled with an AM 1.5 global filters (100 mW/cm^2^). For visible light measurement, a long-wave pass filter (λ > 430 nm) was placed in front of the light source.

## Additional Information

**How to cite this article**: Park, J. *et al.* Structural Evolution of Chemically-Driven RuO_2_ Nanowires and 3-Dimensional Design for Photo-Catalytic Applications. *Sci. Rep.* **5**, 11933; doi: 10.1038/srep11933 (2015).

## Supplementary Material

Supplementary Information
