# Corrigendum: Structural Evolution of Chemically-Driven RuO2 Nanowires and 3-Dimensional Design for Photo-Catalytic Applications

**DOI:** 10.1038/srep16138

**Published:** 2015-11-04

**Authors:** Joonmo Park, Jae Won Lee, Byeong Uk Ye, Sung Hee Chun, Sang Hoon Joo, Hyunwoong Park, Heon Lee, Hu Young Jeong, Myung Hwa Kim, Jeong Min Baik

Scientific Reports 5: Article number: 11933; 10.1038/srep11933published online: 07 07 2015; updated: 11 04 2015

The original version of this Article contained a typographical error in the spelling of the author Sung Hee Chun which was incorrectly given as Sung He Chun.

In addition, the original version of the Article did not indicate that Myung Hwa Kim and Jeong Min Baik jointly supervised the work.

These errors have been corrected in both the PDF and HTML versions of the paper.

