# Supplementary material for: Structural Evolution of Chemically-Driven RuO2 Nanowires and 3-Dimensional Design for Photo-Catalytic Applications
Source: Sci Rep. 2015 Jul 7;5:11933. doi: 10.1038/srep11933 (PMC4493639; doi:10.1038/srep11933)

Supplementary information

Structural Evolution of Chemically-Driven RuO_2 Nanowires and 3-Dimensional Design for Photo-Catalytic Applications

Joonmo Park[1], Jae Won Lee[1], Byeong Uk Ye[1], Sung He Chun[2], Sang Hoon Joo[3], Hyunwoong Park[4], Heon Lee[5], Hu Young Jeong[6], Myung Hwa Kim[2]* & Jeong Min Baik[1]*

[1]School of Materials Science and Engineering, KIST-UNIST-Ulsan Center for Convergent Materials, Ulsan National Institute of Science and Technology (UNIST), Ulsan 689-798, Republic of Korea.

[2]Department of Chemistry & Nano Science, Global Top 5 Program, Ewha Womans University, Seoul 120-745, Republic of Korea.

[3]School of Energy & Chemical Engineering Department of Chemistry Ulsan National Institute of Science & Technology (UNIST), Ulsan 689-798, Republic of Korea.

[4]School of Energy Engineering, Kyungpook National University, Daegu 702–701, Republic of Korea.

[5]Department of Materials Science and Engineering, Korea University, Anam-dong 5-ga, Seongbuk-gu, Seoul 136-713, Republic of Korea.

[6]UNIST Central Research Facilities (UCRF), Ulsan National Institute of Science and Technology (UNIST), Ulsan 689-798, Republic of Korea.

Corresponding author: jbaik@unist.ac.kr

Figure S1. The length and diameter of RuO_2 nanowires with the annealing temperature.

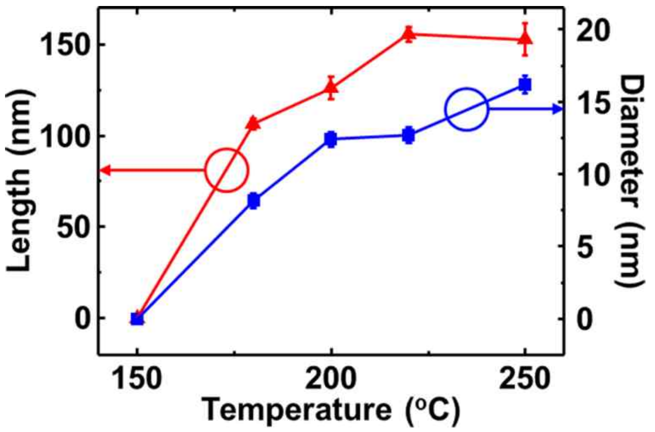

Figure S2. (a) Schematic diagram of electrochemical cell for voltammetry. (b) Schematic energy level diagram of plasmonic system with OEC for solar water splitting.

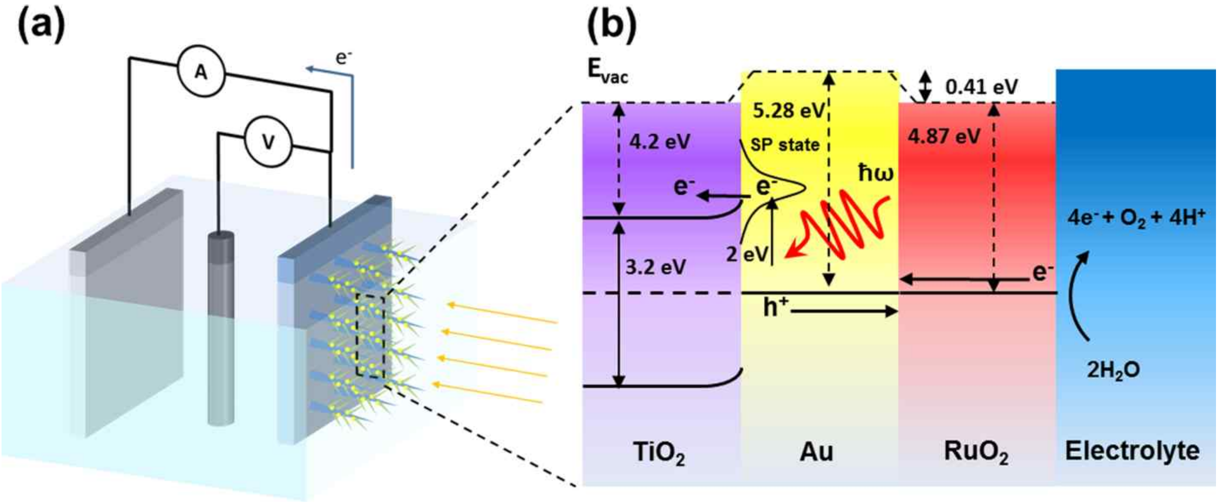

Supplement: Supplementary Information [file srep11933-s1.pdf]
